# Quantifying multi-dimensional attributes of human activities at various geographic scales based on smartphone tracking

**DOI:** 10.1186/s12942-018-0130-3

**Published:** 2018-05-09

**Authors:** Xiaolu Zhou, Dongying Li

**Affiliations:** 1 Department of Geology and Geography, Georgia Southern University, 68 Georgia Ave, Herty Bldg 0201, Statesboro, GA 30460 USA; 2 Yunnan University of Finance and Economics, Longquan Road 237, Kunming, 650221 Yunnan China; 3 Department of Landscape Architecture and Urban Planning, Texas A&M University, College Station, TX 77843 USA

**Keywords:** Individual activity tracking, Location-based step, Smartphone, Tile systems, Geographic information systems

## Abstract

**Background:**

Advancement in location-aware technologies, and information and communication technology in the past decades has furthered our knowledge of the interaction between human activities and the built environment. An increasing number of studies have collected data regarding individual activities to better understand how the environment shapes human behavior. Despite this growing interest, some challenges exist in collecting and processing individual’s activity data, e.g., capturing people’s precise environmental contexts and analyzing data at multiple spatial scales.

**Methods:**

In this study, we propose and implement an innovative system that integrates smartphone-based step tracking with an app and the sequential tile scan techniques to collect and process activity data. We apply the OpenStreetMap tile system to aggregate positioning points at various scales. We also propose duration, step and probability surfaces to quantify the multi-dimensional attributes of activities.

**Results:**

Results show that, by running the app in the background, smartphones can measure multi-dimensional attributes of human activities, including space, duration, step, and location uncertainty at various spatial scales. By coordinating Global Positioning System (GPS) sensor with accelerometer sensor, this app can save battery which otherwise would be drained by GPS sensor quickly. Based on a test dataset, we were able to detect the recreational center and sports center as the space where the user was most active, among other places visited.

**Conclusion:**

The methods provide techniques to address key issues in analyzing human activity data. The system can support future studies on behavioral and health consequences related to individual’s environmental exposure.

## Background

The interaction between human activities and the environment has been an important topic in many disciplines, such as transportation research, urban planning, and public health [4, 15, 29–31]. An increasing number of studies over the past decades have aimed to achieve better understandings of the environmental and health consequences related to travel behavior. Among these studies, tasks such as collecting and processing data related to human activities are mostly laborious and time-consuming. Several challenges exist in collecting and processing human dynamic data.

Regarding data collection, technology advancements have spurred new ways of recording activity data. Many studies have used wearable devices such as global positioning system (GPS) receivers and accelerometers to measure people’s activity space and physical activities [22, 26, 27]. Although activity-tracking devices are becoming more affordable, logistical challenges still exist in carrying out experiments, especially when large sample sizes are needed. Wearing additional devices such as an accelerometer or GPS is cumbersome for participants. In recent years, with rapid growth in smartphone ownership [21], studies have explored using smartphones as sensors to measure travel behavior [19, 31]. However, most studies have focused on total steps averaged across a day based on the daily step counting apps. There is much less discussion on location-based steps, i.e., registering steps to different locations. Issues such as battery consumption and positioning accuracy in covered areas are considered challenging when using smartphone as a tool to track movements [1, 11].

Even for the studies that have considered location-activity interaction, point locations and point density are usually the only information captured as the characteristics of the activities or the individuals’ activity space. Data that are critical to understanding the activity, such as the intensity and duration of the activity that occurred in different locations are usually missing. Another missing piece of information in the empirical studies using smartphone-tracked activity is how accurate the point locations are at various locations. This piece of information is vital because positioning can be less precise in covered areas or urban canyons, resulting in finding that may be less reliable.

Second, in studies analyzing activities, data regarding environmental features are often examined as correlates. Conventional approaches rely on official and commercial datasets, which demonstrate issues of data availability, hierarchy and subjectivity. Nowadays, crowd-sourcing geographic data, such as OpenStreetMap (OSM) are increasingly detailed and accessible. As approaches to collectively produce knowledge, OSM offers new ways in unfolding the fine-scale phenomena that are usually neglected by official datasets. If smartphone-based tracking can be integrated and analyzed with OSM data easily, researchers could gain greater access to rich data regarding a broad range of place of interests (POIs). In addition, OSM uses hierarchical tile systems (i.e. a grid system that controls the level of detail a map should display) to store and display geographic information. The strength in the tile system is that data are extracted and prepared at different scales, boosting the computerational efficiency. In order for the human activity research community to take advantage of the rich OSM dataset, new approaches that can process activity data and link them with OSM data become critical.

To fill these gaps, we proposed and implemented an innovative system that integrated smartphone-based step tracking and the sequential tile scan techniques to collect and process activity data at various spatial scales. For data collection, the system involved using smartphones as mobile sensors to collect location-based steps. We used a smart sensor coordination approach to track users’ locations and steps. Such an approach can improve the previously reported limitations in using smartphone as an activity tracker. For data processing, we proposed the sequential tile scan technique to calculate the activity routes and the associated attributes (i.e. probability, step, and duration surfaces). We applied OSM tile system to aggregate the positioning points at variable scales. The duration surface represents the duration of activity in every tile. The step surface measured the spatial distribution of steps and displayed the tiles that were associated with more steps. The probability surface estimated how accurate the location logs were in every tile. With this approach, researchers can readily link a multifold of activity data, e.g., steps during physical activity, duration of stay in a certain area, with OSM information at various scales. In the following sections, we introduce related studies, explain the system in detail, and present an example application of the approach.

## Related studies

### Monitoring individual’s environmental exposure in behavioral research

Prolonged inactivity poses a critical, life-long health risk. A growing body of evidence indicates that built environment characteristics influence lifestyles. For example, people who live in neighborhoods that are close to stores, recreational places, green spaces, playgrounds, walkways, and bike paths participate in more active travel and have a lower body weight [6, 12, 29].

Previous studies point out that one challenge for healthy behavior research is to contextualize people’s spatial behavior and activities [16]. Many studies used predefined boundaries such as census blocks [17, 23], or various sized buffer areas around points of interest, such as home location [13] to represent activity spaces. However, neither boundaries nor buffer areas around a point of interest capture the actual environment where physical activity takes place. In fact, most people’s physical activities happen in a broader range of space beyond their residential neighborhood. The environmental features where physical activity takes place may also differ from those in the residential neighborhoods [27, 29]. A review found that 90% of the studies in the area of physical activity and environment measured environmental characteristics of participants’ residential neighborhoods and that only 4% of studies examined nonresidential locations [18]. To identify the environmental characteristics that promote active behaviors, it is critical to overcome this limitation by using actual location-based data.

The limitation exists in studies analyzing people’s dietary environment. Many studies have computed dietary environment based on pre-defined areal units or buffer areas around individuals’ home addresses [7]. However, the measured environment may be very different from where individuals choose to eat. For example, when one drives along the highway, the neighborhoods close by are not easy to access despite the proximity. The uncertainty of contextual influences that individuals experience calls for a detailed activity data and multi-facet business information to ensure the rigor of the measured environment [10].

### Smartphone-based tracking

Issues related to GPS devices and accelerometers in activity tracking involve the cumbersomeness in wearing two sets of devices, technical difficulties, and time commitment [30, 31]. In comparison, smartphone tracking demonstrated fewer participant dropouts and greater capabilities in incorporating acceleration values [16]. As a result, smartphones have been used as “urban sensing” devices and are used in studies examining travel and activity patterns [24].

Despite its advantages, smartphone tracking is not without limitations [9]. Recording accuracy, especially with signal losses in indoor areas and areas with dense foliage or buildings, has been a challenge for all types of GPS tracking [3]. In addition, step counting has become common in different smartphone operating systems. Most modern smartphones are equipped with motion sensors. For Android, since API level 19 (KITKAT), most Android phones provide step counter that tracks the number of steps taken by users. For iOS, since iPhone 5 s, similar sensors have been used to record daily steps. Experiments have reported that smartphone-based step detectors perform as accurately as most wearable devices [8]. However, beyond the summary number of total step count, applications to record steps along with the location information on a continuous base have been lacking.

### Shifting scales and the OSM tiles systems

In the past two decades, the environmental health research community has more attention on disaggregated and individual scales. Regarding GIS-based analysis, the traditional uneven access to data and tools between experts and local citizens poses important hurdles. Public participation geographic information systems (PPGIS) initiatives were proposed to combat such unevenness [14, 25]. As an innovative type of PPGIS, the OpenStreetMap takes inputs and edits from certified users, who usually are locals with knowledge about their environments. The data collected for OSM are at the most disaggregated, individual scale and can support inquiries at a variety of levels. To assist flexible data output, the tile system is used so that data can be presented based on the zoom level specified by the user.

This approach has huge potentials in analyzing and visualizing individual travel behavior. These strengths call for a platform that could integrate activity location collection, storage, visualization, and analysis using the OSM tile systems. Based on the tile system, data and analysis can be dynamic and flexible.

## Methods

### Location-based steps

In this study, we used the Android platform to implement the location-based steps. Both motion sensors and position sensors were applied in our program. For position sensors, devices used GPS or network (cellular tower or WiFi access points) to determine the locations. Table 1 shows the strengths and limitations of of the three types of the positioning sensors [28], based on which we developed our system using a hybrid-positioning scheme to detect location-based steps.

**Table 1:** The strengths and limitations for each of these sensors [28]

Techniques	Spatial accuracy (m)	Battery Consumption	Limitations
GPS (A-GPS)	8	High	Indoors or high rise areas
WiFi	74	Low	Areas without access points
Cell-Id	600	Very low	Regions without cell signals

We used several techniques to reduce the battery cost, such as sensor rotation and activity controlled switch [32]. Generally, sensor rotation aims to rotate network-based and GPS-based positioning. The activity controlled switch turns on location sensor when people start moving while turns off the location sensor when people become still. In this study, we developed two modules to control the sensors, i.e., control module and sensor module. Location and step information from the sensor module is sent to the control module every 30 s. The control module determines the sensor statuses and sends commands back to the sensor module. We set the frequency of location updates to be 10 s and 10 m in minimum displacement between location updates if positioning sensor is switched on. Outputs of the program included movement trajectories and steps in continuous space and time dimensions.

### OSM tile system

**Fig. 1:**
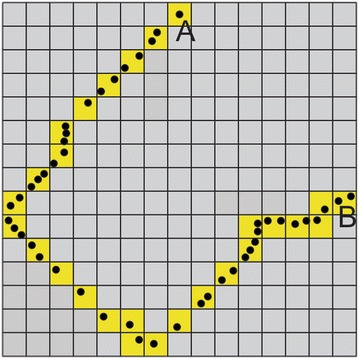
Raster space for trajectory. Points represent trajectory of a person moves from origin *A* to destination *B*. Yellow grid shows the grids overlapped with positioning points

Once the steps and movement data were collected, the next step was to construct the activity space and associate activities with the environment. Currently, OSM and some other mapping service providers, such as Google Maps, offer rich geographic information. It would be ideal to link location-based steps with their tile systems directly. One approach would be directly converting positioning points into grids. Tools such as Points to Grid are available in most GIS programs to achieve such a conversion. However, it would be redundant to store every grid when no activity falls in the grid (grey grids in Fig. 1). The storage redundancy is especially significant if the raster covers a wide area with high resolution but the actual activities happen sparsely. The following sections introduce our proposed method.

In our system, we used a tile system that modeled activity data using a system similar to the OSM tile system. The OSM tiles that are associated with different zoom levels are predefined for the entire earth. Therefore, we set up the positioning points with the tiles at different zoom level directly. There are three advantages to set up the activity data display and analysis system in such a way. First, instead of creating a grid of tiles within the rectangular extent defined by all the positioning points, we only needed to identify the tiles that intersected with the positioning points. This process largely addressed the storage redundancy problem. Second, because the tile systems were associated with multiple zoom levels, it was fast and easy to switch research granularity at different spatial scales. For instance, for the same dataset, higher zoom levels (e.g., zoom 22) could be selected when the research question was at the scale of urban streets, while lower zoom level (e.g. zoom 18) could be used when there was a need to aggregate the data to a larger area. Third, tiles from the OSM offered rich geographic information (e.g., stores, parks, and road networks). Such geographic information can be directly tied to the activity data to analyze the association between human behavior and the environmental context.

The OSM use a Spherical Mercator Projection coordinate system. The tile system includes tiles in a hierarchical structure based on zoom levels. For instance, at zoom level 0, the world is represented by 1 tile while at zoom level 2, the world is divided into 16 tiles. Tile number is determined by $N = 2^{n} \times 2^{n}$, where n is the zoom level [20].

To convert positioning points to tile IDs, the following equations were applied to the latitude and longitude pairs [20].

$$\begin{aligned} & L = 2^{n} \\ & IDx = Math.floor\left( { L * \left( {\frac{{\left( {lon + 180} \right)}}{360}} \right)} \right) \\ & IDy = Math.floor\left( {L*\frac{{1 - \frac{{Math.\log \left( {Math.\tan \left( {Math.toRadians\left( {lat} \right)} \right) + \frac{1}{{Math.\cos \left( {Math.toRadians\left( {lat} \right)} \right)}}} \right)}}{Math.PI}}}{2}} \right) \\ \end{aligned}$$

where *lat, lon* are the coordinates*, toRadians* converts angle degrees to radians.

### Mapping positioning points to the tile system

Once we converted points into the tile system, we need to determine the path that connected adjacent positioning points to form activity routes. In computer graphics, the Bresenham’s line algorithm [5] provides an efficient way to form a close approximation of straight-line grids between two given points. In this project, we extended Bresenham’s line to construct the activity routes.
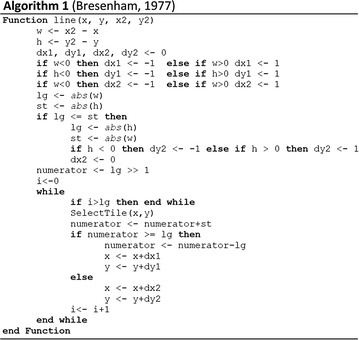


**Fig. 2:**
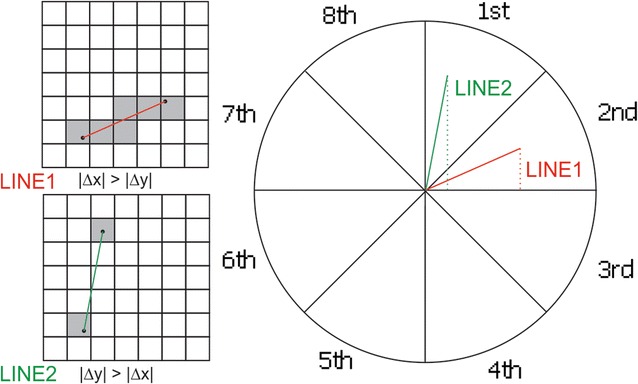
Creation of the path that connecting adjacent positioning points to form activity route. Lines in different octants need different treatment to form continuous route

Suppose we used LINE1 to link two adjacent positioning points and we aimed to find the tiles intersect with such line. If we scanned tiles horizontally, when |∆x| ≥ |∆y| (LINE1), the y-axis increment associated with one unit x-axis increase would be less than one. This process would produce continuous tiles. However, when |∆y| ≥ |∆x| (LINE2), the above-mentioned equation would generate disconnected tiles (Fig. 2). Hence, we considered conditions in different octants. In addition, when calculating the slope, using floating-point data type and considering infinitely large slope would decrease the efficiency. Therefore, we used the Bresenham’s line algorithm to simplify the line tile intersection problem by only using integer variables and removing the costly division operation for slope calculation (Algorithm 1). This process was efficient in generating straight-line grids between two given points.

### Step and duration surfaces

After mapping points to the tile system and connecting tiles with straight-line grids, we computed the number of steps and activity duration within each tile. We assumed uniform motion between two adjacent points, which was reasonable with a short sampling interval. Hence, we divided the steps and duration by the tile numbers between the adjacent positioning points. The results were assigned to each tile. Because the sum of the probabilities of all the tiles along each perpendicular scan equaled to one, the total numbers of steps (or duration) was calculated as $S_{total} = \mathop \sum \nolimits_{i}^{range} \mathop \sum \nolimits_{j}^{seq} p_{i} \times s_{j}$, where *range* is the width of the possible tiles on the perpendicular direction, *seq* is the total number of tiles between the two points, and *p* and *s* are the probability score and step number allocated to each tile.

### Probability surface

**Fig. 3:**
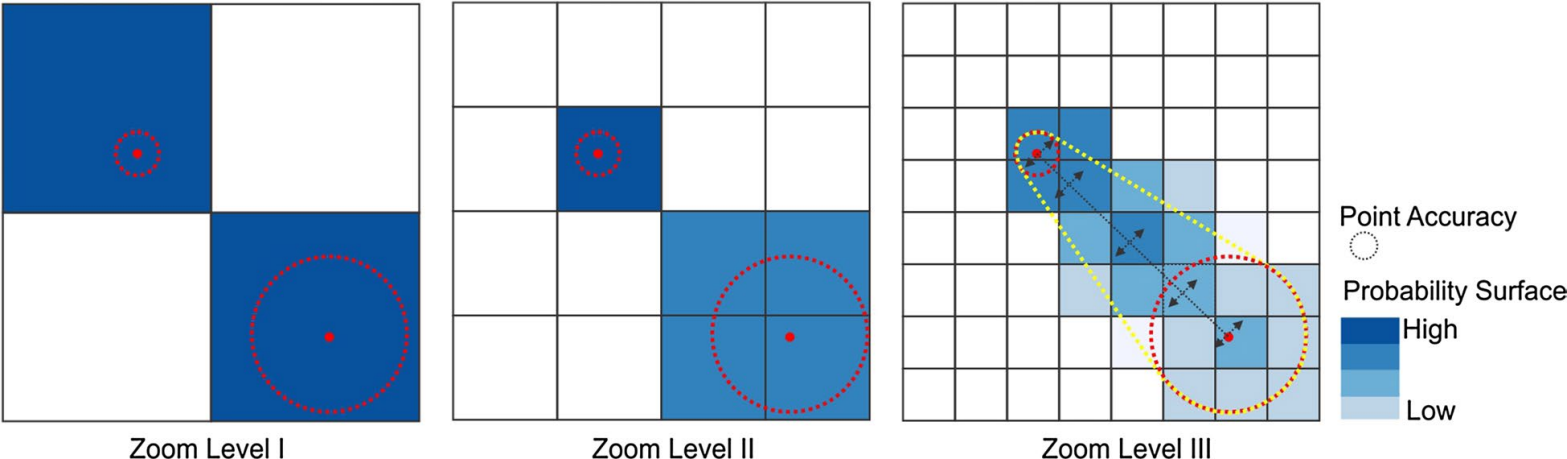
Probability surfaces at different zoom level for the same positioning points and accuracy

We also estimated the activity probability surface. The accuracy of the positioning points could vary from meters to thousand meters. Therefore, quantifying the probability of a point locating in a certain tile was important. The app reported both the locations and the estimated accuracy range. The red points on Fig. 3 were the measured positioning points, and the dashed circles were the accuracy ranges. When the accuracy range was smaller than the tile resolution, the person was very likely located inside the tile. When we increased the zoom level, the certainty of person in a particular tile decreased. In addition, the tiles along the route that connected two adjacent points became more uncertain. In this study, we assumed that people were more likely to move in a straight line between two adjacent points. The assumption was especially safe with short intervals (e.g. 30 s). It was also reasonable to assume that the probabilities decreased when the distance increases away from the central straight line. For instance, at Zoom Level III in Fig. 3, tiles intersected with the straight line between two points had the highest present probability. The probabilities gradually fell as the tiles moved away from the central straight line.

**Fig. 4:**
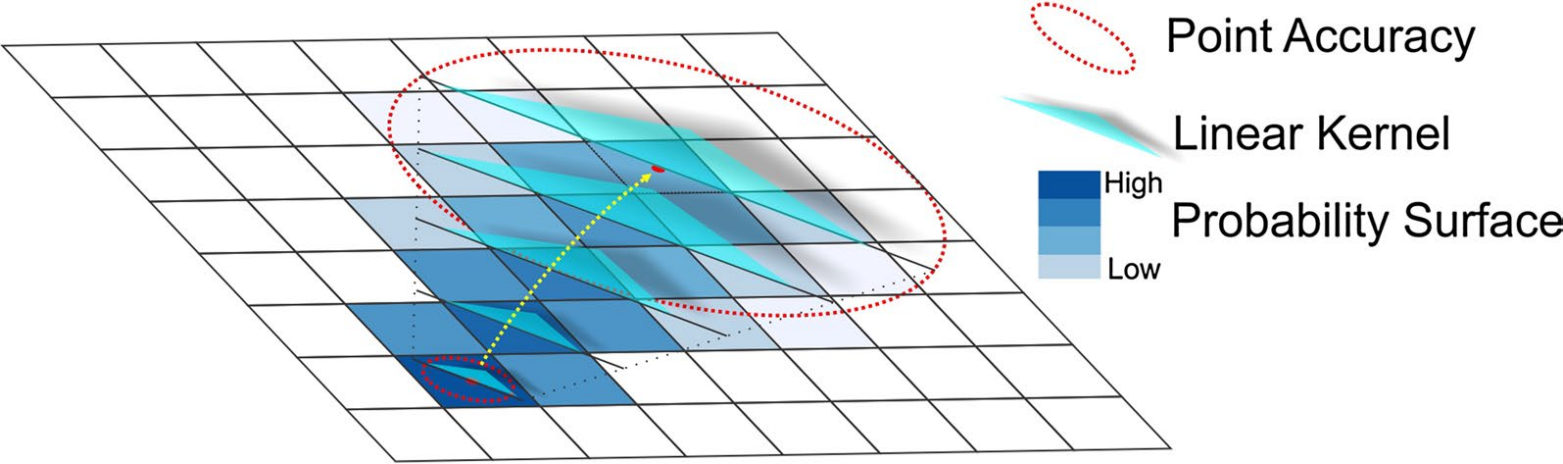
The process to compute the probability surface connecting the adjacent two positioning points

To calculate the probability surface, one way is to find the bounding polygons for the possible activity space (the yellow polygons on Fig. 3) and intersect with the tile system. However, it is computationally expensive to calculate bounding intersection. In this study, we used sequential tile scan technique to calculate the probability surface. This technique involved four steps. We first calculated the possible tile range for the two consecutive positioning points based on the tile resolution and the accuracies of the positioning points. This step helped to determine the numbers of tiles to be considered surrounding the positioning points. Second, we computed the tile sequence connecting the positioning points using Bresenham’s line algorithm. Third, we scanned through all tiles sequentially to create perpendicular tiles. The widths of the perpendicular tiles were determined by the tile range of the current center tile. Fourth, an inverse proportional function was applied to each perpendicular tile to calculate the present probability scores for each tile. This step guaranteed that probability scores decreased when tiles were away from the central straight lines and the sum of the probabilities of all tile along the perpendicular line equaled one (Fig. 4).
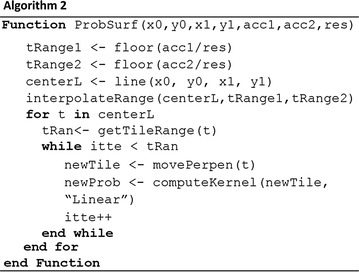

### Extracting place of interest from OSM

After detecting when and where people were active at multiple geographic scales, we needed to extract environmental associates of human activities from OSM. OSM provides rich and free geographic data available across the world. The tags along with geographic data also bring additional information, such as names, addresses, and land use type to complement activity information. We first extracted POIs from all OSM tags. The POI set was based on OSM Map Feature List (https://wiki.openstreetmap.org/wiki/Map_Features). We applied OsmPoisPbf (https://github.com/MorbZ/OsmPoisPbf) to filter geographic features and stored them in PostGIS database. The tags in key-value pairs were stored as hstore data type. Second, we conducted spatial queries in PostGIS to select geographic features that overlap with activity tiles and appended the tag information from OSM to the end of corresponding activity tiles.

### Application of the system in public health

There is a growing interest in developing new ways to measure personal activity and determine the environmental features that are relevant to the behavioral patterns and health consequences. Dietary environment is one of the topics.To demonstrate the effectiveness of our system within the context of human’s dietary environment, we tested the application for 5 days, aiming to capture information about physical activity and dietary space. In the result section, we present the performance of the application in capturing and integrating information such as steps, activity duration, locations, and patterns generated by examining the smartphone and OSM datasets.

## Results

### App interface

**Fig. 5:**
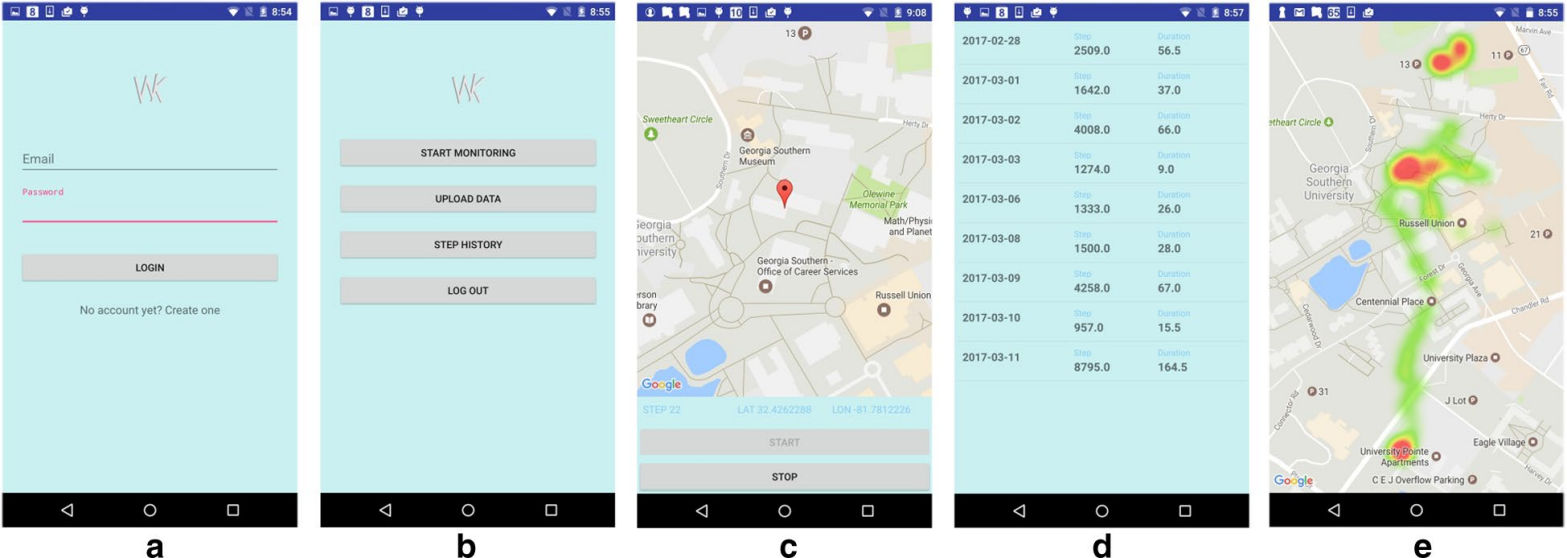
The main interfaces of the application. **a** Login page. **b** Main menu. **c** Monitoring page. **d** Daily activities. **e** Step surface

Figure 5 shows the main functionalities of the application. The interface is easy-to-follow and self-explanatory. We focused on the main functions and techniques that coordinated location and motion sensors rather than interface design in this project. Screen (a) shows the login page. Screen (b) shows the main menu. It allows users to start or stop monitoring, upload data, and look up previous location-based steps. Screen (c) is the monitoring page; the instantaneous steps and locations are updated on the map and the information section at the bottom of the page. Screen (d) shows a list of recorded steps and activity durations for each day. Once a record is selected, Screen (e) will display the step surface.

### Multi-dimensional attributes of travel behaviors

To demonstrate the effectiveness of the application in capturing multi-dimensional attributes of travel behaviors, one researcher used the app for 5 days and analyzed the trajectories and relevant information. The resulting test data include a sum of 43.11 h of information. We collected 2245 point locations, translating to 1053 tiles at level 19. The positioning accuracy ranged from 3 meters to 2900 m, with a median accuracy of 12 meters. In our project, the system generated three layers to represent the activity space: activity step surface, duration surface, and probability surface.

#### Step surface

**Fig. 6:**
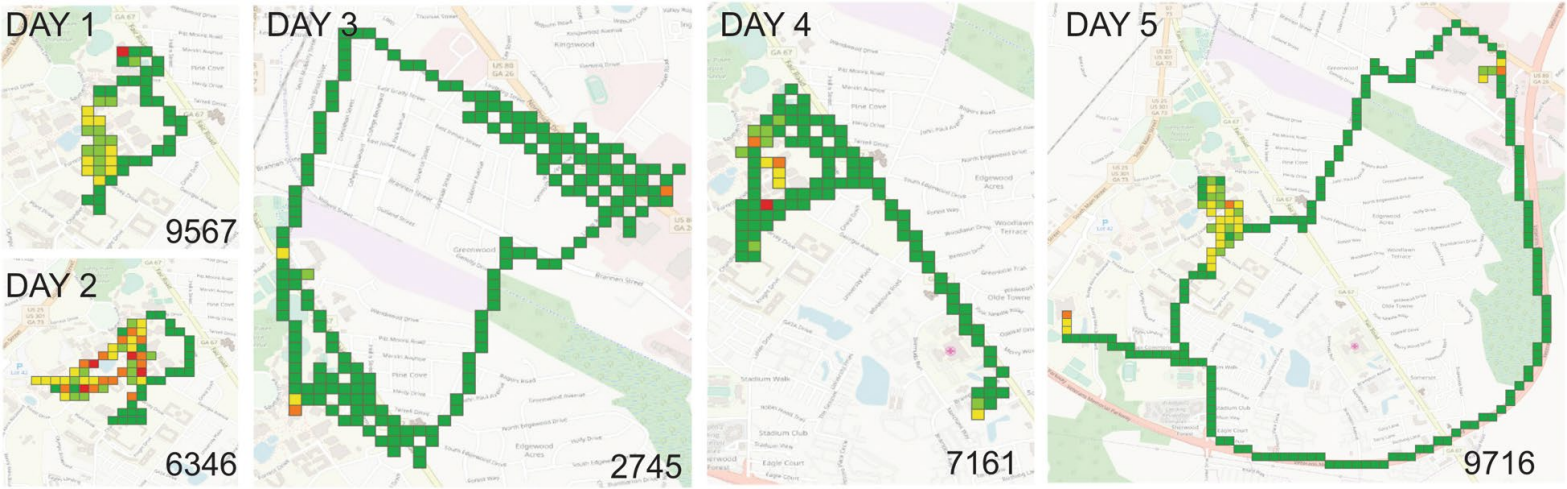
Moving trajectories plotted at Zoom Level 19 in 5-day case study. Number at the bottom represents the total step number for the day

The step surface demonstrated the locations where the user actually walked. People’s activity space may cover a broad area, but the places where people actually walked can be very limited. For the step surface, the value associated with each tile is the actual steps accumulated in the corresponding tile. Figure 6 shows the distribution of moving trajectories at zoom level 19. The colors in the map represent the number of steps: the green end of the color ramp representing fewer steps and the red end of the color ramp representing more steps. Numbers at the bottom present the daily step counts. From this surface, we could identify the locations where steps concentrated.

#### Duration surface

**Fig. 7:**
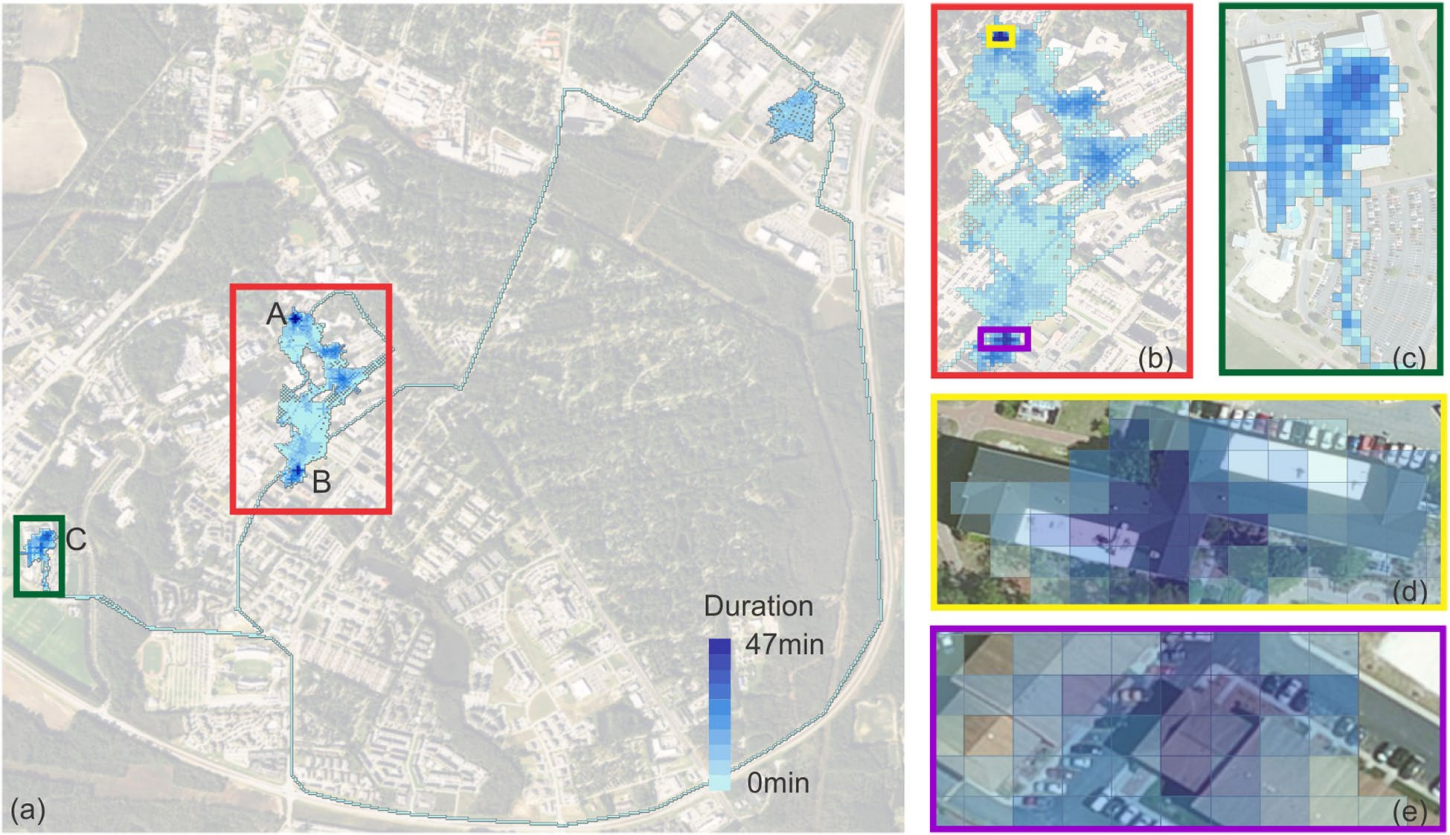
The duration surface of a user’s activities. Tiles with darker color represent longer stay time. Two places with significantly longer stay duration are at Spot A and B. **b** shows the enlarged map for the red box on **a** and **c** shows the enlarged map for the green box on **a**. **d**, **e** show the detailed locations for spot A and B receptively

In addition to the step surface, we also produced the duration surface to map where and how long people stay in each area in their daily life. By doing so, we could find anchor points (e.g. home or workspace) for users’ daily activities. To better illustrate the duration surface, we plotted the travel behavior on Day 5 at a higher zoom level (Zoom Level 22). Figure 7 shows the duration surface. The longest duration of activity within a tile is 47 min. Among all activity tiles, we can spot two places (Spots A and B) with higher duration value. These two places were the home and workplace for the user. Spot C also emerged as an important destination. In addition, the tile system at level 22 displays very detailed ground information about the locations visited. For example, Fig. 7d, e show the buildings where the individual spent most of his/her time.

#### Probability surface

**Fig. 8:**
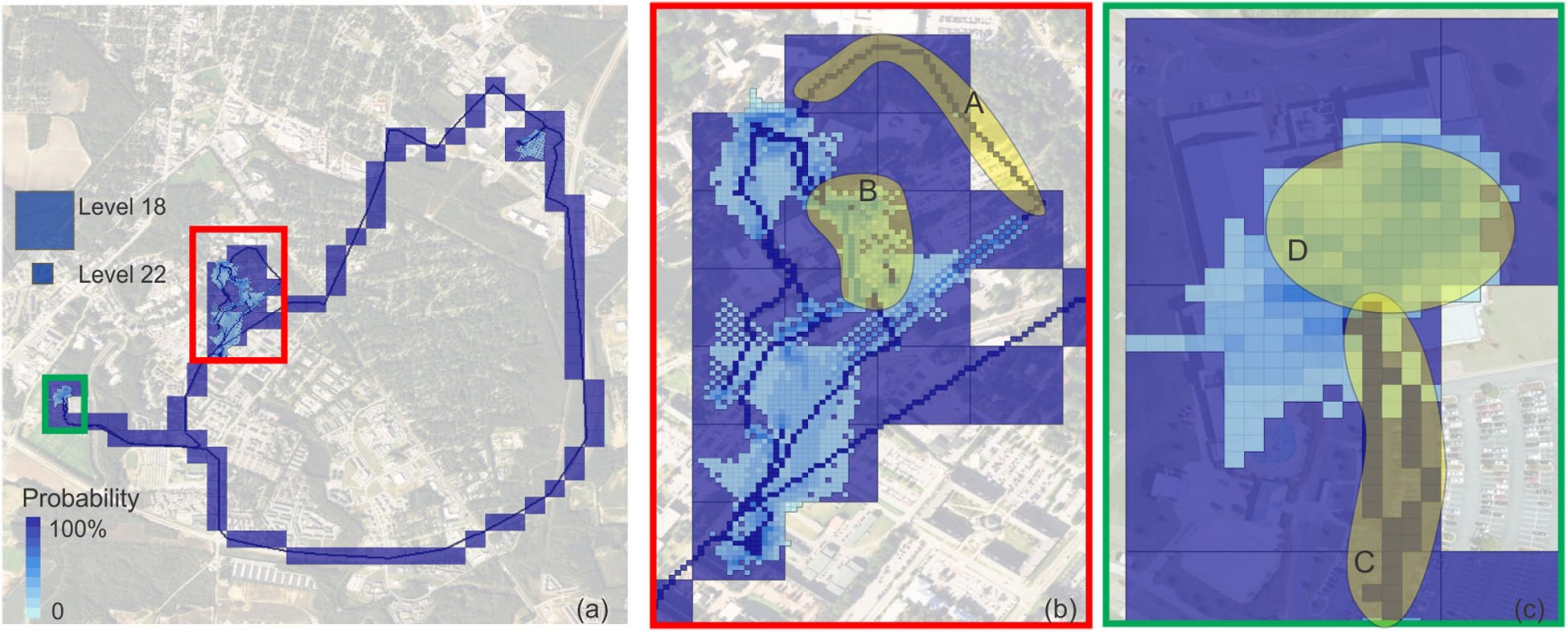
**a** The probability surface of a user’s daily activities. Activities at two tile levels (Level 18 and 22) are displayed. The probability ranges from 0 to 1, with darker color representing higher probability. **b** is the enlarged map for red box area on **a**. **c** is the enlarged map for green box area on **a**

Probability surface was generated to show the accuracy of the locations. Figure 8 shows the probability surface of the individual’s daily activities. We used two zoom levels (i.e. Level 18 and 22) to reflect the probability surface on Day 5. At Level 18, the resolution for each tile was about 128 meters in this area. Most tiles at this zoom level had a probability of 100%, which suggested that we were very positive that the individual had their footprints on these tiles. At level 22, the resolution for each tile was about 8 meters in the study area. This level provided detailed movement information. In addition, when the positioning accuracy was high, we could acquire very certain estimation about the actual movement path (such as route A). On the other hand, when the positioning accuracy was relatively low (lower than the resolution level), we still could map out the possible path and the associated probability scores (such as area B). Figure 8c shows the contrast between outdoor and indoor activities. GPS provided high positioning accuracy outdoors, so the certainty of tiles was very high (Area C). When using network positioning inside the building, although the overall accuracy was lower, we could still map out the most possible activity space (Spot D) in the building by using the probability surface.

#### POI detections

**Fig. 9:**
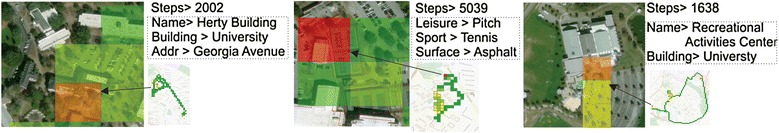
POIs, corresponding tags, and steps numbers at the most active places in the 5-day moving trajectory. Information in the dashed boxes is derived from OSM tags

With these in-depth examinations of individual’s route, steps, and duration of stay, we could investigate the environmental associates of the travel behaviors. Using the OSM tile system, travel behavior were directly linked with points of interests (POIs) provided by OSM data. This feature supported visual assessment and further spatial statistical analyses in examining the correlation between characteristics of destinations and physical activity. To show the corresponding POIs where high number of steps concentrated, we queried the OSM database to detect POIs. Figure 9 shows the most active places and their POI information. We identified that the user was most active in the recreational activities center, tennis courts, and the Herty Building. The corresponding steps at these places were also displayed.

**Fig. 10:**
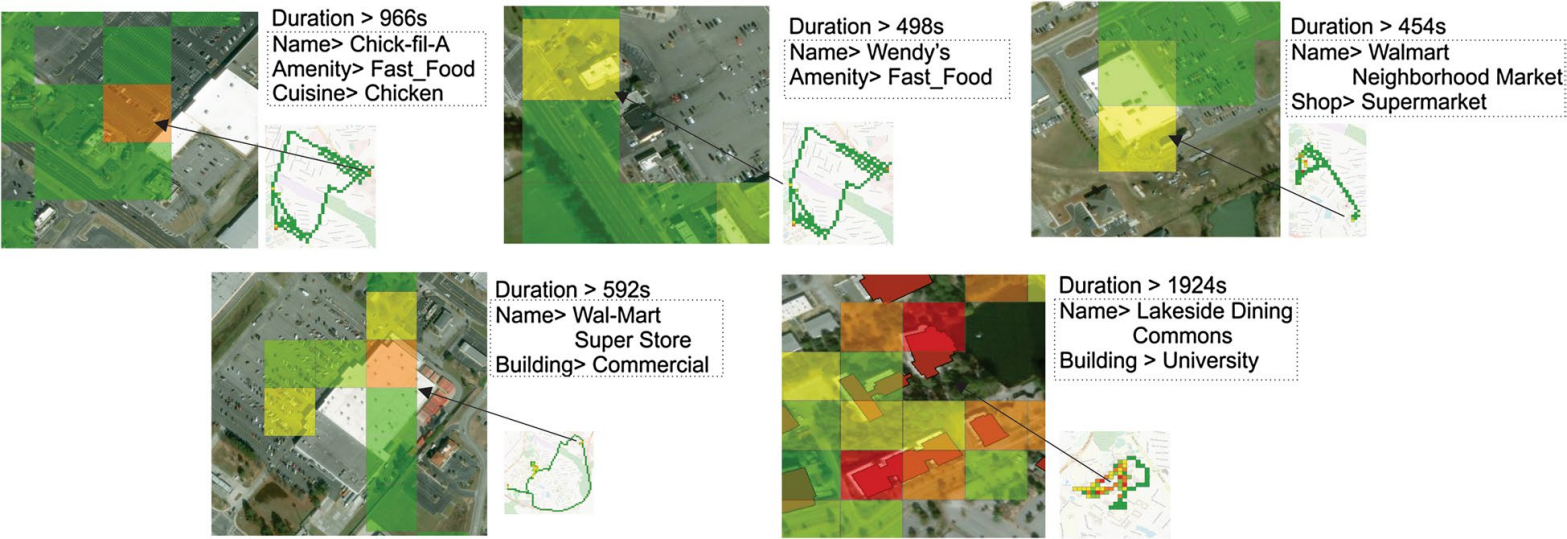
POIs, corresponding tags, and durations for the dining and grocery activities in the 5-day case study

Activities happen in restaurants or grocery stores are important indicators in analyzing people’s dietary behaviors. We integrated the trajectory data with OSM data to detect dining or grocery shopping activities. POIs in tiles related to food and grocery were selected if trajectories intersected with those tiles for more than 5 min. Figure 10 shows the POIs related to dining and grocery places the user visited in the 5-day case study. From the POIs, we found that the user visited at least two fast food venues (Chick-fil-A and Wendy’s) and two grocery stores.

#### Change between spatial scales

**Fig. 11:**
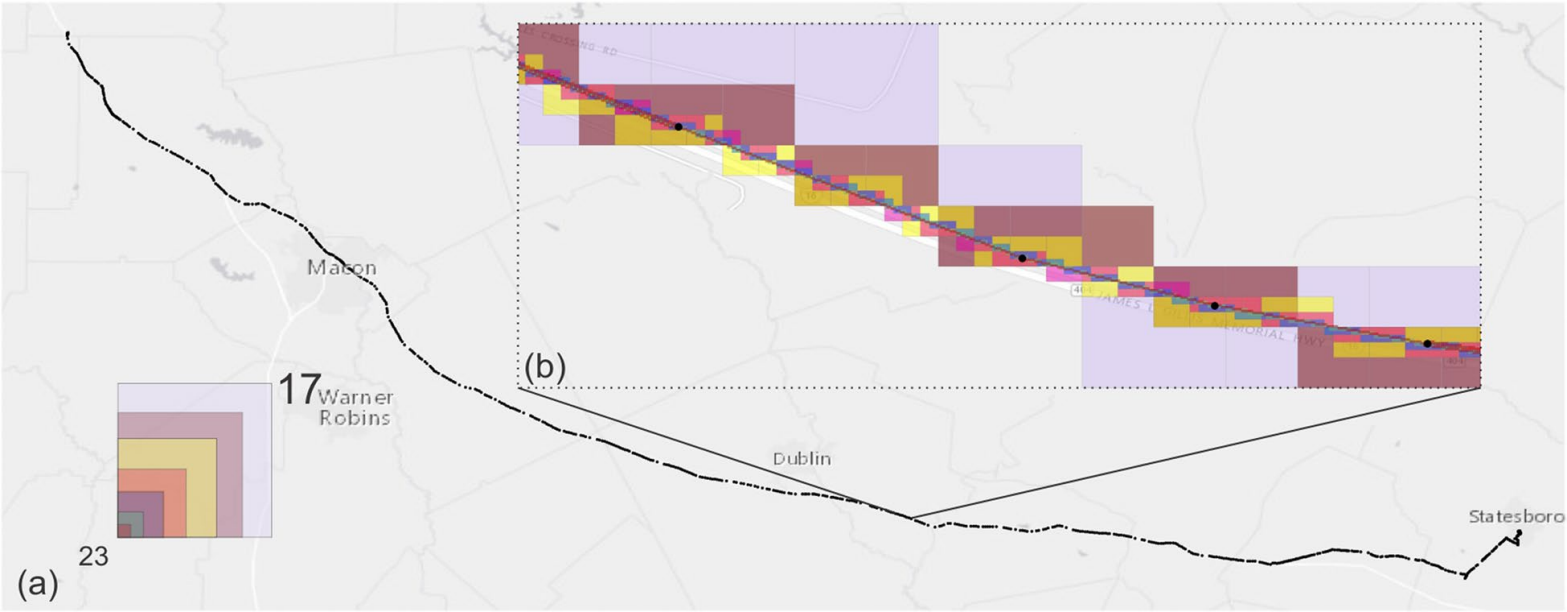
**a** GPS point distribution along a high way. **b** An enlarged map to show tile size at different zoom levels from 17 to 23

Flexible and agile methods to process activity data across multiple scales help facilitate activity data analysis. In order to evaluate the performance of our methods to process activity data across multiple scales, we collected a larger scale dataset with GPS positions to test the algorithm. Figure 11 shows the user continuously drove for 164.3 miles on the highway while using the app. Each black dot on the figure represents one positioning point. We collected 1003 points along this route. Using this dataset, we compared the computation time, resolution, and tile numbers generated for multiple zoom levels. Figure 11b shows an enlarged road segment, which shows the tiles at zoom levels from 17 to 23. The higher the zoom level, the better the tiles fit the actual route. When the zoom level is greater than 21, the generated tiles can mostly represent the actual route.

Table 2 shows the process time in milliseconds, tile resolutions in meters, and the generated tile numbers associated with each zoom level. At zoom level 22, the system generated 42,562 tiles in 3.4 s on a computer with 16 GB memory and Intel i7-4770 at 3.4 GHz processor. A resolution of 8 meters was high enough for most applications and it was very fast to generate these tiles.

**Table 2:** Process time, resolution, and tile numbers that corresponding to different zoom level

Zoom level	Process time (MS)	Resolution (m)	Tile number
17	1190	256.42	1954
18	1229	128.21	2880
19	1338	64.105	4722
20	1561	32.05	8497
21	1998	16.03	16,751
22	3408	8.01	42,562
23	15,230	4.00	178,946

To summarize the results, the sequential tile scan technique can estimate the step, duration and probability surfaces efficiently, which is very useful in quantifying multi-dimensional attributes of the activity spaces. We applied OSM tile system to aggregate the positioning points at various scales. Activity spaces were compatible with OSM tile systems, which facilitated geographic data collection and examination along with the human activity data.

## Discussion

Conceptually, this study offers a new way of examining and aggregating individual GPS trajectories. First, this platform supports research that examines individual’s travel trajectories. By focusing on daily activity places instead of the arbitrary residential neighborhood, this approach represents improvements in capturing the environmental exposure associated with behavioral and health outcomes. Moreover, based on the collected positioning points, we computed a continuous probability surface to quantify the likelihood of individuals being at certain locations.

Another methodological contribution of this study is to tie multi-dimensional attributes, e.g. the step and duration surfaces with travel patterns. In human–environment research, methods have been proposed to delineate the contextual areas to which individuals expose based on GPS points, but limited attention has been paid to identifying walking behavior and recording the duration of activities in specific environmental context. As a result, the entire activity space is usually given equal consideration when analyzing the environmental associates of behavior. However, as we know from previous research, the health and behavioral outcomes vary based on type of activity, intensity, and duration [2]. Our approach provides data regarding steps and duration of activity in the different tiles that could be examined in relation to different environmental characteristics.

Also, the aggregation process helps safeguard the privacy issue associated with personal location data. Researchers and individual users can select to release data at the level where private information cannot be traced, which is particularly useful in generating visualizations to show the spatial patterns of travel behavior.

Finally, this proposed platform combines a number of GPS data collection, visualization and analysis functionalities for research and everyday use. The technical issues regarding transforming and converting data across platforms present challenge for researchers and limits the use of GPS tracking in a variety of research. Our platform, however, supports processes that involves the collection of individual data, aggregation based on specific research goals and analytical needs.

There are several limitations in this study. First, because the possible tiles that connect origin and destination were interpolated based on the consecutive location points, locations where the signal is weak or the participant is sedentary were not recorded. Therefore, the data has to be cleaned and incorrect trajectory records needed to be removed. In future studies, we will incorporate the road network data and map matching methods to address such situations. Second, the comprehensiveness of OSM data varies in different cities. When analyzing the activities along with OSM data, the richness of the findings rely heavily on detailed information regarding POIs. In the future, we can combine geographic data from multiple sources to increase the coverage of POIs in cities. Third, because our focus of this project was to develop the functionality to capture multi-dimensional attributes of activities, we did not deploy our application to a larger group of participants. Instead, we only obtained a test dataset by running the app for 5 days. The application was able to capture useful information at multiple scales. We also demonstrated the captured information could be used in projects in measuring physical activities and environmental exposure. In the future, we plan to conduct a comprehensive assessment. We will recruit participants and invite them to use the app for an extended period. Along with the app, we will also use pedometers and accelerometers and self-reported activity diaries to validate the app-based step and duration measures. We also plan to apply the proposed system in empirical studies investigating issues regarding the human–environment interaction, such as crime risk modeling and food exposure analysis.

## Conclusion

To conclude, in this study, we proposed and implemented a system that integrates smartphone-based step tracking and the sequential tile scan techniques to collect and process activity data at various scales. Such a system can be applied to a number of human dynamics studies to achieve better understandings of the behavioral and health consequences related to individual’s environmental exposure.

## References

[1] Abdulazim T, Abdelgawad H, Habib K, Abdulhai B (2013). Using smartphones and sensor technologies to automate collection of travel data. Transp Res Rec: J Transp Res Board.

[2] Barton J, Pretty J (2010). What is the best dose of nature and green exercise for improving mental health? A multi-study analysis. Environ Sci Technol.

[3] Bellad V, Petovello MG, Lachapelle G. Intermittent tracking in weak signal environments. Paper presented at the Indoor Positioning and Indoor Navigation (IPIN), 2015 International Conference on (2015).

[4] Boarnet M, Crane R (2001). The influence of land use on travel behavior: specification and estimation strategies. Transp Res Part A: Policy Pract.

[5] Bresenham J (1977). A linear algorithm for incremental digital display of circular arcs. Commun ACM.

[6] Carroll-Scott A, Gilstad-Hayden K, Rosenthal L, Peters SM, McCaslin C, Joyce R, Ickovics JR (2013). Disentangling neighborhood contextual associations with child body mass index, diet, and physical activity: the role of built, socioeconomic, and social environments. Soc Sci Med..

[7] Caspi CE, Sorensen G, Subramanian SV, Kawachi I (2012). The local food environment and diet: a systematic review. Health Place..

[8] Case MA, Burwick HA, Volpp KG, Patel MS (2015). Accuracy of smartphone applications and wearable devices for tracking physical activity data. JAMA.

[9] Chen R. Introduction to smart phone positioning. Ubiquitous positioning and mobile location-based services in smart phones. Hershey, PA: IGI Global, 1–31; 2012.

[10] Chen X, Kwan MP (2015). Contextual uncertainties, human mobility, and perceived food environment: the uncertain geographic context problem in food access research. Am J Pub Health..

[11] Cottrill C, Pereira F, Zhao F, Dias I, Lim H, Ben-Akiva M, Zegras P (2013). Future mobility survey: experience in developing a smartphone-based travel survey in Singapore. Transp Res Rec: J Transp Res Board.

[12] Duncan MJ, Winkler E, Sugiyama T, Cerin E, Leslie E, Owen N (2010). Relationships of land use mix with walking for transport: do land uses and geographical scale matter?. J Urban Health..

[13] Feng J, Glass TA, Curriero FC, Stewart WF, Schwartz BS (2010). The built environment and obesity: a systematic review of the epidemiologic evidence. Health Place..

[14] Haklay M (2013). Citizen science and volunteered geographic information: overview and typology of participation. Crowdsourcing geographic knowledge.

[15] Handy S (1996). Methodologies for exploring the link between urban form and travel behavior. Transp Res Part D: Transp Environ.

[16] Kerr J, Duncan S, Schipperjin J (2011). Using global positioning systems in health research: a practical approach to data collection and processing. Am J Prev Med.

[17] Koohsari MJ, Badland H, Giles-Corti B (2013). (Re)Designing the built environment to support physical activity: bringing public health back into urban design and planning. Cities..

[18] Leal C, Chaix B (2011). The influence of geographic life environments on cardiometabolic risk factors: a systematic review, a methodological assessment and a research agenda. Obesity Rev..

[19] Nitsche P, Widhalm P, Breuss S, Brändle N, Maurer P (2014). Supporting large-scale travel surveys with smartphones: a practical approach. Transp Res Part C: Emerg Technol.

[20] OSM. (n.d.). Slippy Map Tilenames. from http://wiki.openstreetmap.org/wiki/Slippy_map_tilenames.

[21] PRC. (2015). Mobile Fact Sheet. from http://www.pewinternet.org/fact-sheet/mobile/.

[22] Quigg R, Gray A, Reeder AI, Holt A, Waters DL (2010). Using accelerometers and GPS units to identify the proportion of daily physical activity located in parks with playgrounds in New Zealand children. Prev Med.

[23] Riva M, Gauvin L, Barnett TA (2007). Toward the next generation of research into small area effects on health: a synthesis of multilevel investigations published since July 1998. J Epidemiol Community Health..

[24] Shin D, Aliaga D, Tunçer B, Arisona SM, Kim S, Zünd D, Schmitt G (2015). Urban sensing: using smartphones for transportation mode classification. Comput Environ Urban Syst.

[25] Sieber R (2006). Public participation geographic information systems: a literature review and framework. Ann Assoc Am Geogr..

[26] Stewart OT, Moudon AV, Fesinmeyer MD, Zhou C, Saelens BE (2016). The association between park visitation and physical activity measured with accelerometer, GPS, and travel diary. Health Place.

[27] Troped PJ, Wilson JS, Matthews CE, Cromley EK, Melly SJ (2010). The built environment and location-based physical activity. Am J Prev Med.

[28] Zandbergen PA (2009). Accuracy of iPhone locations: a comparison of assisted GPS, WiFi and cellular positioning. Trans GIS.

[29] Zenk SN, Schulz AJ, Matthews SA, Odoms-Young A, Wilbur J, Wegrzyn L, Stokes C (2011). Activity space environment and dietary and physical activity behaviors: a pilot study. Health Place.

[30] Zhou X, Li D, Larsen L (2016). Using web-based participatory mapping to investigate children’s perceptions and the spatial distribution of outdoor play places. Environ Behav.

[31] Zhou X, Yu W, Sullivan WC (2016). Making pervasive sensing possible: effective travel mode sensing based on smartphones. Comput Environ Urban Syst.

[32] Zhuang Z, Kim K-H, Singh JP. Improving energy efficiency of location sensing on smartphones. Paper presented at the Proceedings of the 8th international conference on Mobile systems, applications, and services; 2010.

